# Euo is a developmental regulator that represses late genes and activates midcycle genes in *Chlamydia trachomatis*

**DOI:** 10.1128/mbio.00465-23

**Published:** 2023-08-11

**Authors:** Owais R. Hakiem, Syed M. A. Rizvi, Cuper Ramirez, Ming Tan

**Affiliations:** 1 Department of Microbiology and Molecular Genetics, University of California Irvine, Irvine, California, USA; 2 Department of Developmental and Cell Biology, University of California Irvine, Irvine, California, USA; 3 Department of Medicine, University of California Irvine, Irvine, California, USA; Pennsylvania State University, University Park, Pennsylvania, USA

**Keywords:** gene expression, gene regulation, regulon, RNA-seq, DNA immunoprecipitation

## Abstract

The pathogenic bacterium *Chlamydia* reproduces via an unusual intracellular developmental cycle in which it converts from a dividing form (reticulate body or RB) to an infectious form (elementary body or EB). The transcription factor Euo has been proposed as a developmental regulator in *Chlamydia trachomatis* because it repressed a number of late chlamydial promoters, which are transcribed during RB-to-EB conversion. To define the Euo regulon, we performed a genome-wide study that combined Euo DNA immunoprecipitation-seq (DIP-seq) studies with RNA-seq analysis of HeLa cells infected with an Euo-overexpressing *C. trachomatis* strain. We demonstrate that Euo directly regulates ~7% of *C. trachomatis* genes. However, only about half were downregulated (28/61; 45.9%) by Euo overexpression while paradoxically the other half were upregulated (33/61; 54.1%). Intriguingly, all downregulated genes were late genes, while the majority of upregulated genes were midcycle genes, which are transcribed during RB replication. DIP analysis showed that Euo occupancy sites were restricted to the core promoter region for downregulated genes but were located over a wider region immediately upstream of the promoter for upregulated genes. We also found that Euo controls its own expression through a negative feedback mechanism. Electron microscopy analysis of cells infected with the Euo-overexpressing strain showed fewer EBs, consistent with a block in RB-to-EB conversion, as well as fewer and larger RBs. Together, these findings broaden the role of Euo as a developmental regulator that functions as both a transcriptional repressor of late genes and a transcriptional activator of midcycle genes in *C. trachomatis*.

**IMPORTANCE:**

In this study, we developed a correlative approach that combined DNA immunoprecipitation-seq and RNA-seq analyses to define the regulon of the *Chlamydia trachomatis* transcription factor Euo. We confirmed the proposed role of Euo as a transcriptional repressor of late chlamydial genes but also showed that Euo activates transcription of a subset of midcycle genes and autoregulates its own expression via negative feedback. This study validates and expands the role of Euo as an important developmental regulator in *C. trachomatis*. In addition, this genome-wide correlative approach can be applied to study transcription factors in other pathogenic bacteria.

## INTRODUCTION

Chlamydiae are obligate intracellular bacteria that convert between two specialized forms during an unusual biphasic developmental cycle. An infectious form called an elementary body (EB) binds and enters a eukaryotic host cell within a membrane-bound vacuole called the chlamydial inclusion (1 – 3). Within a few hours, the EB differentiates into a reticulate body (RB), which is the metabolically active but non-infectious form. The RB then divides repeatedly, accompanied by RB size reduction, and the bacterial population inside the host cell expands (3, 4). Starting at 24 h post infection (hpi) for *Chlamydia trachomatis*, RBs begin asynchronously converting into EBs. The *C. trachomatis* intracellular infection ends at about 48 hpi with host cell lysis, or extrusion of the inclusion, which releases EBs to infect new host cells (5). RB division and RB-to-EB conversion are thus critical steps in the chlamydial developmental cycle that are necessary for a productive infection.

A central feature of this developmental cycle is the coordinated expression of chlamydial genes in three main temporal waves (6, 7). Early genes are transcribed during the time of EB-to-RB conversion, while the majority of chlamydial genes are transcribed in midcycle during RB replication. Late genes are a subset of chlamydial genes that are of particular interest because they are upregulated at the time of RB-to-EB conversion, starting at about 24 hpi. A few late genes have known EB-specific functions (8), such as *hctA* and *hctB*, which encode histone-like proteins HctA (Hc1) and HctB (Hc2) that condense DNA in EBs (9, 10), and *omcAB*, which encodes two EB-specific outer membrane proteins (11). However, the roles of many late genes have not been established, although they are presumed to be involved in RB-to-EB conversion or EB function.

Late genes have been proposed to be regulated by a *Chlamydia*-specific transcription factor called Euo (12, 13). Euo is expressed as early as 1 hpi, and *euo* transcript levels can be detected throughout the developmental cycle, although there is experimental disagreement about whether transcript levels remain relatively constant (6), or are higher at early time points (14). *C. psittaci* Euo preferentially bound DNA at adenine and thymine (AT)-rich sequences, although a specific binding sequence has not been defined (15). Intriguingly, *C. trachomatis* Euo has been shown to bind and repress selected promoters for late genes but not midcycle genes (12). These findings have led to a model in which Euo selectively represses late genes, which prevents their premature expression until repression is relieved at late times by an undefined mechanism (8, 12). This model has yet to be validated in chlamydiae, and the full cohort of Euo-regulated genes that make up its regulon has not been defined. It is also not known if Euo is the sole or major mechanism for regulating the timing of late gene expression in *Chlamydia*.

A number of global approaches are being used to study bacterial transcription factors and their target genes. RNA-seq is widely used to map the regulon of a transcription factor but does not distinguish between directly and indirectly regulated genes. In contrast, DNA-immunoprecipitation (DIP) (16) identifies binding sites for a transcription factor in the genome but does not necessarily demonstrate if binding leads to transcriptional control. In this study, we combined these two genome-wide methods to identify the regulon whose transcription is directly controlled by Euo in *C. trachomatis*. We mapped Euo binding sites with DIP-seq and then performed an RNA-seq analysis of an Euo-overexpressing strain to identify differentially expressed genes (17, 18). Using this combined approach, we provide experimental evidence that Euo is a major transcriptional repressor of late genes in *C. trachomatis*. However, we also discovered that Euo also functions as a transcriptional activator that upregulates the expression of a subset of midcycle genes. In addition, Euo autoregulates its own expression through a negative feedback mechanism. Furthermore, the Euo occupancy pattern in the core promoter region appears to be different for downregulated and upregulated genes, consistent with different mechanisms of transcriptional regulation. To explore Euo function further, we investigated the effect of Euo overexpression on the chlamydial developmental cycle.

## MATERIALS AND METHODS

### Plasmids, strains, and antibodies

All cloning was done using NEBuilder HiFi DNA Assembly kit and the parent plasmid pBOMB4 (19). P*^tet^-euo* (pMT1918) plasmid was constructed by replacing the mCherry gene with the *C. trachomatis euo* coding sequence (CDS) C-terminally fused with 6× His and cloning the *C. trachomatis omcA* promoter upstream of GFP (green fluorescent protein) in the plasmid pBOMB4. The control plasmid (pMT1917) was constructed similarly, except that mCherry was not replaced with *euo* (Fig. 2A, right panel). Polyclonal rabbit antiserum raised against recombinant MBP (maltose-binding protein)-Euo (anti-Euo) was used for all immunoprecipitation (10 µL) and immunofluorescence (1:1,000) experiments. Mone) reads mapped to monoclonal mouse anti-MOMP (major outer membrane protein) antibodies (LifeSpan Biosciences, Shirley, MA, USA) were used for immunofluorescence (1:1,000). Donkey anti-mouse (Alexa Fluor 488, cat# A21202, 1:1,000) and goat anti-rabbit (Alexa Fluor 594, cat# A11037, 1:1,000) were acquired from Invitrogen. Mouse monoclonal anti-GroEL1 antibody (A57-B9) was kindly provided by the Morrison Lab (20) and was used at a dilution of 1:1,000.

### Cell culture and *Chlamydia* infection

HeLa cell line was purchased from ATCC and cultured at 37°C and 5.0% CO_2_ in Dulbecco’s Modified Eagle Medium (DMEM) (Gibco) supplemented with 10% fetal bovine serum (FBS). Cells were grown in T-150 flasks (Falcon cat# 355001) and 6-well plates (Corning cat# 3506) and were infected in 6-well plates for DIP-seq and 24-well plates (Corning cat# 3527) for RNA-seq.

*C. trachomatis* L2 434/Bu strain (ATCC) was used for all experiments. Monolayers of HeLa cells were infected with the Test or Control strain at a multiplicity of infection (MOI) as indicated for each experiment. All infections were done in SPG (sucrose phosphate glutamate) buffer (200 mM sucrose, 20 mM sodium phosphate, and 5 mM glutamate, pH 7.2). HeLa monolayers in 6-well/24-well cell culture plates were overlaid with SPG containing EBs, and plates were centrifuged at 750 × *g* for 1 h at 4°C. After centrifugation, the SPG was removed and replaced with DMEM supplemented with 10% FBS and 1 µg/mL cycloheximide.

### Transformation of EBs

EBs were transformed with the pMT1918 (P*^tet^-euo*) or pMT1917 plasmid to construct the “Test” and “Control” strains, respectively. Transformation was done as described elsewhere (21) with modifications. 1.3 × 10^7^ inclusion forming units (IFUs) of EBs were mixed with 5–10 µg of plasmid DNA in 50 µL CaCl_2_ buffer (10 mM Tris, pH 7.4, and 50 mM CaCl_2_) and incubated for 30 min at room temperature. The mixture was then diluted with 12 mL SPG and used to infect a 6-well plate of nearly confluent HeLa cells (i.e., ~2 mL of the suspension per well). Monolayers were infected [passage 0 (P_0_)] at room temperature by centrifugation for 1 h at 1,000 × *g*, after which SPG was replaced with DMEM containing 10% FBS (2 mL/well). Cultures were supplemented with cycloheximide (final concentrations: 1 µg/mL) and ampicillin (10 µg/mL) at 12 hpi. Cells from each well were harvested in 1 mL SPG at 42 hpi, disrupted by glass beads, and centrifuged for 10 min at 1,000 × *g* and 4°C. The supernatant was collected and used to infect a new 6-well plate of confluent HeLa cells (P_1_). Immediately after infection, medium containing both cycloheximide and ampicillin was added, and the process of harvesting and infection was repeated until GFP-expressing inclusions were observed (typically at the end of P_2_ or P_3_).

To generate a clonal population, transformants were diluted, and a 6-well plate of confluent HeLa cells was infected with dilutions of EBs and overlaid with media (DMEM, 10% FBS, 10 µg/mL ampicillin, and 10 µg/mL cycloheximide) containing 0.55% sterile low-melting agarose. Agarose layer was allowed to solidify and gently overlaid with media. Infection was continued until isolated plaques were visible. A P_200_ (blunt tip) micropipette was used to pick plaques. This process was repeated one more time with the plaque-cloned EBs to ensure clonal population of the transformants. The clonal population of EBs from the second plaque cloning was expanded and purified. The titer (IFU/µL) of EB stocks was determined for each stock.

### Purification of recombinant Euo

Recombinant *C. trachomatis* Euo containing a 6× His tag at the C-terminus was expressed from the plasmid pMT1919. pMT1919 contains the *C. trachomatis euo* gene cloned in an IPTG (isopropyl β-D-1-thiogalactopyranoside) inducible expression plasmid pET22b (ATCC). *Escherichia coli* strains containing pMT1919 were grown in 1-L LB to an A_600_ of 0.6. Cultures were induced with 100 µM Isopropyl β-D-1-thiogalactopyranoside (IPTG) overnight at 16°C, and cells were harvested by centrifugation. The cell pellet was resuspended in buffer A [20 mM Tris-HCl (pH 8.0), 300 mM NaCl, 1 mM β-mercaptoethanol] containing 10 mM imidazole. The resuspended cells were sonicated and centrifuged at 16,000 × *g* for 30 min. The supernatant was incubated with 1 mL slurry of Qiagen Ni-NTA beads for 1 h at 4°C. The beads were washed with 100 mL of buffer A containing 20 mM imidazole. Recombinant Euo protein was eluted from the column with 2 mL of buffer A containing 250 mM imidazole. Eluted protein was dialysed overnight against 1 L of storage buffer [20 mM Tris-HCl (pH 8.0), 100 mM NaCl, 10% (vol/vol) glycerol]. Dialysed protein was aliquoted and stored at −80°C.

### Western blotting

Purified protein, cell lysates, or pull-down samples were prepared by boiling the samples at 95°C for 5 min in SDS loading dye. Equal volumes of lysates were loaded, resolved by SDS-PAGE, and transferred onto PVDF (polyvinylidene difluoride) membrane. Membranes were blocked with 5% bovine serum albumin (BSA) in 1× Tris-buffered saline containing 0.1% Tween 20. After incubation with primary (anti-Euo, 1:10,000; anti-MOMP, 1:10,000) and secondary antibodies (Alexa Fluor 594 goat anti-rabbit, 1:10,000; Alexa Fluor 488 donkey anti-mouse, 1:10,000), PVDF membranes were imaged on an Amersham Typhoon scanner.

### Euo protein pulldown

HeLa cells were infected with the Test strain at an MOI of 3, and the *euo* gene was induced from 0 hpi using 15 nM anhydrotetracycline (aTc) (ThermoFisher Scientific, Waltham, MA, USA). At 28 hpi, cells were washed with 1× PBS (phosphate buffered saline), and lysates were prepared in 20 mM Tris-HCl (pH 8.0), 150 mM NaCl, and 10% (vol/vol) glycerol. Protein G Dyna beads (30 µL) (Invitrogen) were prepared by incubating with the anti-Euo antiserum for 2 h at 4°C. Anti-Euo antibody-labeled beads were incubated with the infection lysates on an end-to-end shaker overnight at 4°C. Subsequently, the beads were washed thrice with buffer containing 20 mM Tris-HCl, pH 8.0, 150 mM NaCl, and 0.1% NP-40, and Euo protein was confirmed by polyacrylamide gel electrophoresis and Western blot. GroEL1 was used as an input control. The Control strain was also induced from 0 hpi using 15 nM aTc and processed for native Euo pulldown in identical manner. Native Euo pulldown from wt. *C. trachomatis* was performed similarly. All pull-down experiments were done in duplicate.

### Immunofluorescence microscopy

HeLa cells, grown and infected on glass coverslips, were fixed in 100% ice-cold methanol for 5 min. Cells were permeabilized and incubated in a blocking buffer (2% FBS, 0.1% Triton) for 30 min at room temperature. *C. trachomatis* Euo and MOMP were labeled using anti-Euo and anti-MOMP antibodies, respectively. *C. trachomatis* and host cell DNA were stained with DAPI. Coverslips were mounted with an anti-fade mounting solution (VECTASHIELD antifade mounting medium with DAPI from Vector Laboratories). Immunofluorescence microscopy images were acquired on Zeiss Axio Observer microscope, and images were generated with Zen Blue software. Inclusion size was measured by drawing boundaries overlapping the MOMP signal for at least a hundred inclusions for each sample by ImageJ sofware’s free hand selection tool.

### Preparation of cells for TEM

HeLa cells were infected with the Test strain at an MOI of 3, and Euo expression was induced from 0 hpi using 15 nM aTc. At the indicated time points after infection, cells were washed with 1× PBS and detached from the plate using trypsin, centrifuged at 300 × *g* for 4 min, and fixed with 2% paraformaldehyde/2.5% glutaraldehyde (Polysciences Inc., Warrington, PA, USA) in 100 mM sodium cacodylate buffer, pH 7.2 for 2 h at room temperature. Processing and imaging of the samples were performed by the Washington University Center for Cellular Imaging facility. The Control strain induced from 0 hpi using 15 nM aTc was processed and imaged in an identical manner. RBs and EBs were counted using ImageJ software. A dividing RB, as indicated by its dumbbell shape, was counted as one RB.

### Progeny assay

HeLa cells were infected with the Test strain at an MOI of 1, and Euo expression was induced with 15 nM aTc at 0 hpi. At the indicated time points after infection, cells were washed with 1× PBS, which was then replaced with 500 µL cold SPG. Cells were lysed by freezing at −80°C for 30 min, followed by thawing at 37°C for 15 min and vigorous vortexing. Cell lysates were serially diluted in SPG and used to re-infect fresh monolayers of HeLa cells. At 28 hpi, cells were fixed in ice-cold methanol, and the numbers of IFUs were determined via immunofluorescence microscopy using anti-MOMP antibody. The number of inclusions was counted from 10 fields of view observed with a 20× objective. The number of progeny per cell was determined by dividing the total number of infectious progeny (IFU/mL) by the number of host cells present at the start of the infection. The number of host cells was quantified by counting trypsinized cells on a hemocytometer.

### *Chlamydia* quantification

The number of chlamydiae per host was measured by qPCR. Crude total DNA extracts were prepared by collecting infected HeLa cells in 100 µL of sonication buffer [1 mM EDTA, 10 mM Tris-HCl (pH7.5), 0.1% SDS] followed by freeze-thaw at −80°C. The crude extract was then diluted 1:10,000, and the copy number of two chlamydial genomic loci (*omcA* and *scc2* promoter regions) and two host genomic loci (*GAPDH* and *YWHAZ*) was quantified by qPCR (see Supplementary table T1 for primer sequences). Chlamydiae per host was measured by calculating *E*_h_^Ct(host)^/*E*_c_^Ct(chlamydia)^ (*E*_h_: PCR efficiency of host genomic locus, *Ct(host)*: Ct value for host genomic locus, *E*_c_: PCR efficiency for *C. trachomatis* genomic locus, Ct(chlamydia): Ct value for *C. trachomatis* genomic locus).

### EMSA

Oligonucleotides (50 bp) containing the Euo operator of *omcA* and putative operator of *euo* were synthesized (Integrated DNA Technologies [IDT]). The fragments were end labeled with [γ-32P] ATP using T4 polynucleotide kinase (New England Biolabs, Ipswich, MA, USA) and purified from the free label using a mini Quick Spin DNA column (Roche) to use as the *euo* probe. Electromobility shift assay (EMSA) was carried out as described in reference (22) using recombinant *C. trachomatis* Euo. A typical binding reaction in 30 µL contained 10 nM labeled probe, 1 µg salmon sperm DNA, 9 µg BSA, 10% (vol/vol) glycerol in GSA (glycerol salmon sperm albumin) buffer containing 20 mM HEPES, pH 8.0, 20 mM Tris-HCl, pH 8.0, 150 mM NaCl, 1 mM EDTA, 1 mM DTT (dithiothreitol) and the indicated amount of protein. The reaction was carried out at 37°C for 30 min. DNA-protein complexes were resolved on 7% native polyacrylamide gels by electrophoresis in 1× Tris-borate-EDTA buffer at 100 V at 4°C after a pre-run at 100 V for 1 h. After electrophoresis, the gel was dried on Whatman paper and exposed to a phosphorimager screen. The screen was scanned with an Amersham Typhoon scanner. Assays were performed in triplicates.

### *In vitro* transcription assay

Approximately 5 nM plasmid DNA containing the transcription template was incubated with 2 µM recombinant Euo at 37°C for 30 min. Transcription assays were initiated as described previously (23) using 2 µL of *C. trachomatis* RNA polymerase. The transcripts were resolved by electrophoresis on an 8-M urea—6% polyacrylamide gel. The gels were then fixed, dried, and exposed to a phosphorimager plate. The plate was scanned with a Bio-Rad Personal FX scanner. For each plasmid, the transcription assays were performed as a minimum of three independent experiments.

### DNA immunoprecipitation-seq

HeLa cells were infected with *C. trachomatis* at an MOI of 1 and harvested at 36 hpi in sonication buffer [1 mM EDTA, 10 mM Tris-HCl (pH7.5), 0.1% SDS] without cross-linking. Cells were sonicated with a Covaris S220 Focused-ultrasonicator for 3 min to achieve an average fragment size of 150 bp. DNA fragments were purified by adding an equal volume of NucleoSpin Gel extraction buffer to the sonicated lysate and mixed before passing it through the NucleoSpin column. For DIP, the binding reaction was carried out in 30 µL with recombinant *C. trachomatis* Euo (50 ng), sheared *C. trachomatis* DNA (1 µg), 1 µg salmon sperm DNA, 9 µg bovine serum albumin in buffer B [20 mM Tris, pH 7.5, 100 mM NaCl, 10% (vol/vol) glycerol] for 30 min at 37°C. Protein G Dyna beads were incubated separately for 2 h at 4°C in buffer B with 10 µL of anti-Euo antibodies. The DIP binding reaction solution was reconstituted to 500 µL using buffer B and incubated overnight at 4°C on an end-to-end rotor with anti-Euo-coated Protein G Dyna beads. After washing (buffer B with 0.1% Tween 20) and elution at 65°C, enriched DNA was purified using Qiagen PCR clean-up kit. qPCR was performed to measure the enrichment as a percentage of input DNA. DIP-seq libraries were constructed using NEXTflex Chip-Seq Kit (Bioo Scientific, Austin, TX, USA), and supplier protocol was followed. Typically, purified fragments were adenylated, adapter ligated, and PCR amplified using NEXTflex Chip-Seq Kit and NEXTflex barcodes (Bioo Scientific). Size selection was performed using AMPure XP beads (Beckman Coulter). The “Input” and’ “No Euo” control libraries were prepared identically. Libraries were paired-end sequenced to 100 bp read lengths on the Illumina Novaseq 6000 sequencing platform at the UCI-Genomics High Throughput Facility.

### Overexpression of Euo

*C. trachomatis* Euo was overexpressed by adding aTc (15 nM) at the time of infection (0 hpi), and samples were harvested at 16, 28, 32, or 36 hpi for differential RNA-seq experiments. Similarly, aTc was added at 0 hpi, and samples were harvested for genome copy number measurement (16, 20, 24, 28, 32, and 36 hpi) or infectious progeny measurement (16, 24, 28, 32, and 36 hpi).

### RNA extraction and RNA-seq

HeLa cells infected with the Test strain were lysed in TRIzol (250 µL/well in a 24-well plate). Samples were collected in 1.5 mL microcentrifuge tubes, and 100 µL chloroform was added. Samples were homogenized by brief vortexing. After centrifugation, the aqueous layer containing total RNA was loaded on a Qiagen RNeasy Mini Kit column. RNA was DNase treated on the column and eluted in nuclease free water (Invitrogen). After RNA extraction, the samples were analyzed by Qubit to control for any degradation of RNA and samples with RNA integrity number (RIN) values >8. The yield of the RNA samples was measured by Qubit. RNA-seq libraries were prepared using Zymo-seq RiboFree Total RNA Library Kit by Zymo Research, and supplier’s instruction was followed. Libraries were paired-end sequenced to 100 bp length on the Illumina NovaSeq 6000 sequencing platform.

### cDNA synthesis and qPCR

Total cDNA synthesis was performed using iScript cDNA synthesis kit (Bio-Rad Laboratories Inc., Hercules, CA, USA). 1 µg of total RNA and one unit of reverse transcriptase (RT) were used in a 20-µL reaction system for cDNA synthesis at 42°C for 30 min followed by inactivation at 85°C for 5 min. All the qPCR experiments for genome copy number assay were performed on a BioRad real-time PCR machine. The LinRegPCR (v2014.6) qPCR data analysis software was used to measure the PCR efficiency for each target using the amplification plots. There are no well-defined housekeeping genes for *C. trachomatis*, and 16S rRNA expression has been shown to be not stable in *C. pneumonia* (24). mRNA level of *C. trachomatis* genes was first normalized to the bacterial genomic DNA (23s rRNA) to measure the expression level per chlamydia. The target amplicon sequence and primer sets are provided in Supplementary table T5.

### High-throughput sequencing data analysis

Read mapping, peak calling, and differential expression analysis were done using Qiagen CLC Genomics Workbench (v. 21.0.5). For DIP-seq analysis, sequenced reads were mapped to the *C. trachomatis* genome L2 434/Bu strain (accession no: AM884176; https://www.ncbi.nlm.nih.gov/nuccore/AM884176) using high stringency settings (mismatch cost: 2, insertion and deletion cost: 3, length and similarity fraction: 0.8 each, maximum number of hits for a read: 10, and minimum read count fusion table: 5). Peaks were called by using “transcription factor ChIP-seq” tool with a *P*-value cutoff of 0.1. For RNA-seq count matrix was generated by “RNA-seq analysis” tool with the following parameter settings—reference type: genome annotated with genes only, use spike-in controls: no, mismatch cost: 2, insertion cost: 3, deletion cost: 3, length fraction: 0.8, similarity fraction: 0.8, global alignment: no, strand specific: both, library type: bulk, maximum number of hits for a read: 10, count paired reads as two: no, ignore broken pairs: yes. The differential expression statistical analysis was done using the “Differential Expression in Two Groups” function, filtering for average expression for false discovery rate (FDR) correction. The *P*-values reported are FDR *P*-values. The default RNA-seq analysis method in CLC Genomics Workbench software counts fragments per kilobase of transcript per million mapped reads (FPKM) instead of individual reads. This method assigns a read count only to unbroken fragment pairs, removing low-quality read fragments from the analysis.

### Annotation of TSSs and genes associated with a DIP-peak

Transcription start sites (TSSs) were annotated based on our RNA-seq data, and annotations published by Albrecht et al. (25). The annotation from NC_010280 (used by Albrecht et al.) was transferred to AM884176 assembly using the annotation software “liftoff” (26), which uses sequence identity between the two assemblies to mark the CDS. We relied on our RNA-seq data when a TSS could be clearly identified and, when possible, compared to annotations by Albrecht et al. When we could not identify a TSS using our RNA-seq data, we calculated the distance between the TSS and the start codon of the downstream CDS using Albrecht et al. data and marked the TSS upstream of the corresponding gene on the genome assembly we used. We then used the TSS to mark putative transcripts to define operons. All annotated CDSs downstream of a TSS and on the same strand were considered to belong to an operon. Genes were associated with a DIP-peak when the peak was present proximal to the 5′ of corresponding putative transcript. All genes in an operon were associated with the peak associated with the first gene of the operon.

## RESULTS

### Euo preferentially and specifically binds to intergenic regions throughout the *C. trachomatis* genome

We used DIP-seq to identify binding sites for Euo in the *C. trachomatis* genome. We incubated 10–500 ng of purified recombinant *C. trachomatis* Euo-His with sheared DNA obtained from *C. trachomatis*-infected HeLa cells and then used anti-Euo antibodies to immunoprecipitate (IP) Euo and its bound DNA fragments. In proof-of-principle experiments, we used qPCR to verify that this procedure successfully recovered regions of the *C. trachomatis* genome known to bound by Euo *in vitro* (P*^omcAB^* and P*^scc2^*) (12). This analysis showed that P*^omcAB^* and P*^scc2^* were specifically and reproducibly enriched with as low as 10 ng of Euo, while negative control regions of the host genome (*YWHAZ*) and *C. trachomatis* genome (23s rRNA and groES) were not enriched (Fig. S1A through D). This result demonstrated that Euo specifically binds to *C. trachomatis* DNA and is not titrated by the bulk of host DNA *in vitro*.

**Fig 1:**
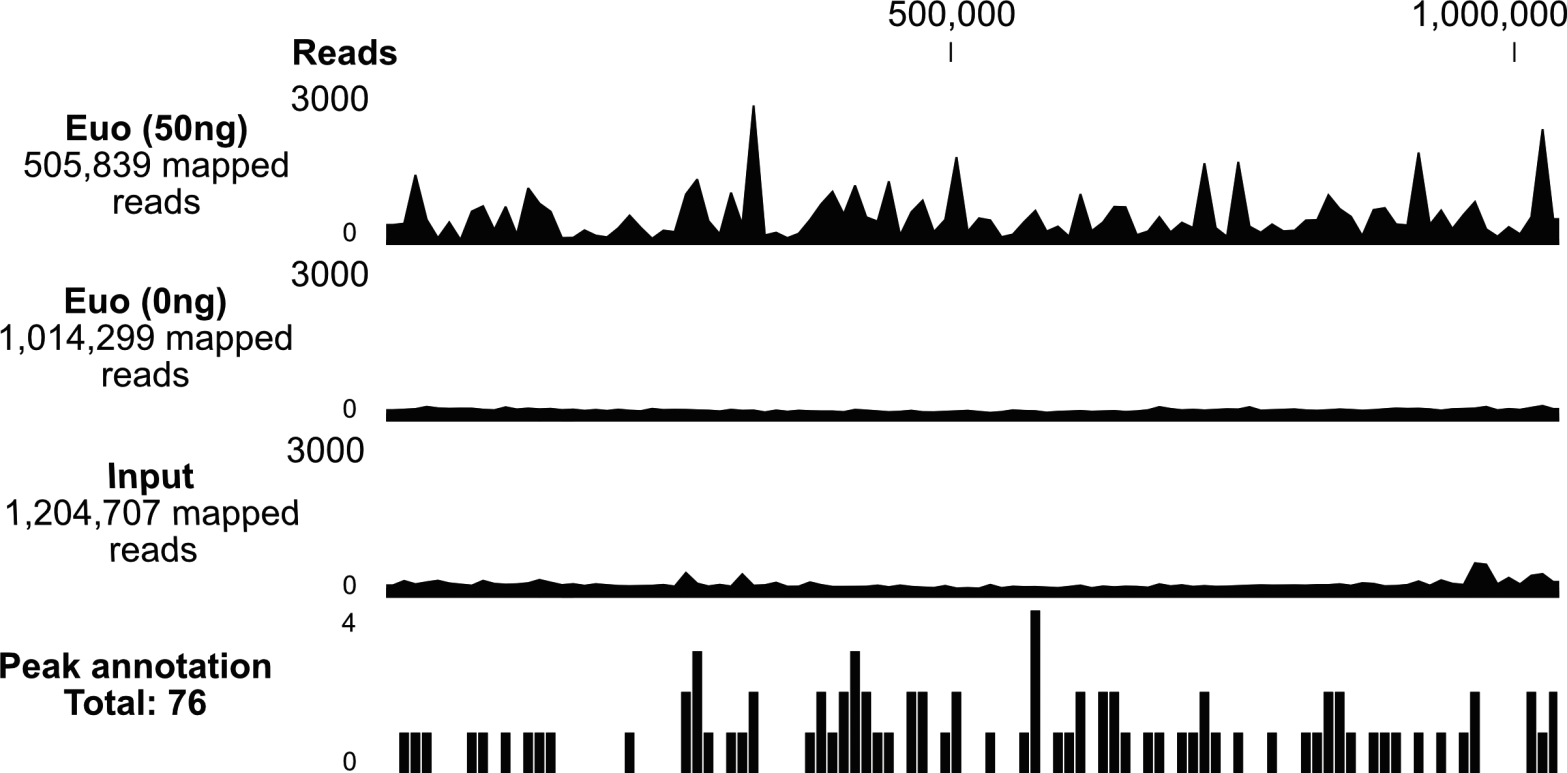
Mapped reads of enriched *C. trachomatis* chromatin after DIP. The left panel shows the quantity of Euo (50 or 0 ng) and input (total DNA before IP [immunoprecipitation]). The bottom panel represents the annotated peaks after comparing read mappings of DIP and input samples (*n* = 2). Link to UCSC Genome Browser: https://genome.ucsc.edu/s/merajrizvi/Chlamydia.

To perform DIP-seq, we repeated this Euo DIP procedure and used the immunoprecipitated DNA to construct libraries for sequencing. Reads were mapped to the *C. trachomatis* serovar L2 genome (see Materials and Methods), and the mapping profile was compared for libraries immunoprecipitated with 10 or 50 ng Euo, 0 ng Euo (mock enriched), and an input DNA library. The read mapping profile was reproducible and consistent for both 10 and 50 ng of Euo (Fig. S1E). The 50 and 0 ng libraries were sequenced for ~102.7 million and ~78.8 million reads, respectively, of which 505,839 (0.49%, 48.7 coverage) and ~1 million (1.29%, 97.6 coverage) reads, respectively, mapped to the *C. trachomatis* genome. The input DNA library was sequenced for ~71.5 million reads, of which ~1.2 million (1.68%, 115.9 coverage) reads mapped to the *C. trachomatis* genome (Fig. 1; Table S1). In the absence of Euo (0 ng), no enrichment was observed (Fig. 1; Fig. S1E).

By comparing the mapped reads for the 50 ng Euo and input libraries, we identified 76 Euo DIP-seq peaks in the *C. trachomatis* genome. The peaks were widely and evenly distributed over the entire genome without any clustering. Based on the annotated coding sequences (27), the majority of these peaks (72/76, ~95%) were in intergenic regions where promoters are likely to be located. The remaining four peaks were located within the CDSs but were within 300 bp downstream of a start codon. These results identified binding sites for Euo within the *C. trachomatis* genome. They also suggest that the large majority of candidate Euo binding sites are in the vicinity of the promoter for the gene immediately downstream of the DIP-peak.

To extend this analysis to genes in an operon that are co-transcribed from the same promoter, we identified predicted operons in the *C. trachomatis* genome. Using TSS data from Albrecht et al. (25) and RNA-seq done in this study, we identified an operon as two or more adjacent genes on the same strand, downstream of the same TSS (details in Materials and Methods). If there was a DIP-peak in the vicinity of the promoter for this putative operon, we labeled all genes in the operon as DIP-peak-associated genes. In total, we classified 197 genes in the *C. trachomatis* genome as DIP-peak-associated genes (Table S2).

### Overexpression of Euo downregulates expression of late genes but also upregulates expression of some midcycle genes

**Fig 2:**
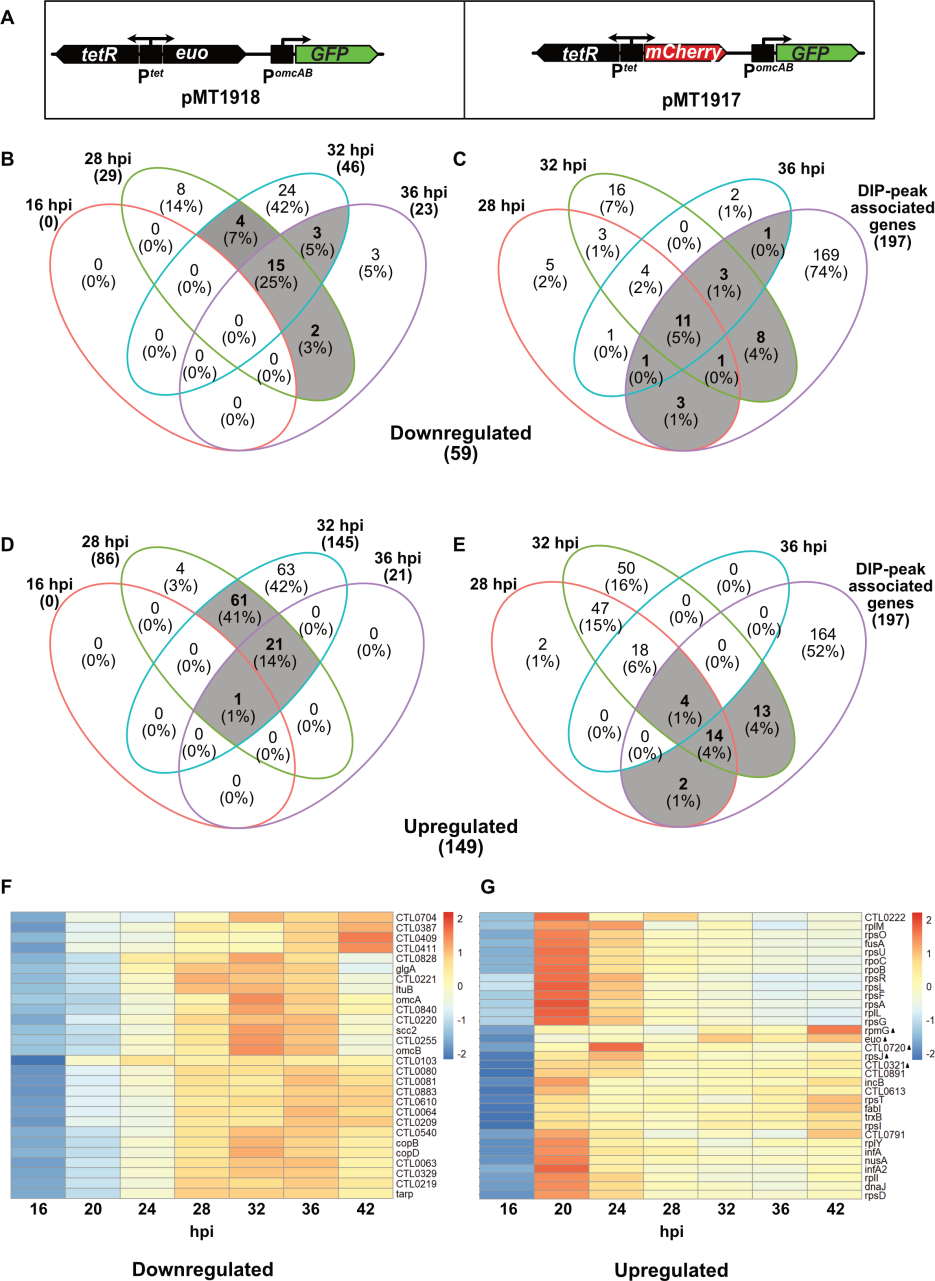
(A) Map of the region of the *euo* overexpression plasmid pMT1918 (left panel) showing that it contains the *C. trachomatis euo* gene cloned under the control of an inducible P*^tet^* promoter and GFP cloned downstream of the *C. trachomatis omcAB* promoter (P*^omcAB^*). In the control plasmid pMT1917 (right panel), *euo* was replaced with the mCherry gene. (B) Venn diagram showing the total number of downregulated genes after 16, 28, 32, and 36 h of Euo overexpression, and the number of downregulated genes common between time points. Numbers in braces are the total number of genes downregulated at each time point. (C) Venn diagram showing the total number of genes downregulated by Euo after 28, 32, and 36 hpi, the total number of genes associated with DIP-peaks and the number of genes common between different categories. (D) Venn diagram showing the total number of upregulated genes after 16, 28, 32, and 36 h of Euo overexpression, and the number of upregulated genes common between different time points. (E) Venn diagram showing the total number of genes upregulated by Euo after 28, 32, and 36 hpi, the total number of genes associated with DIP-peaks and the number of genes common between different categories. Genes with fold change 1.33 and *P*-value 0.05 are considered differentially expressed. (F) Heatmap showing temporal expression pattern of the 28 DIP-peak-associated downregulated genes. (G) Heatmap showing temporal expression pattern of the 33 DIP-peak-associated upregulated genes. Test, Euo overexpressing *C. trachomatis* strain; Control, mCherry expressing *C. trachomatis* strain.

To determine which genes are regulated by Euo, we identified genes that were differentially expressed in response to Euo overexpression. We constructed an Euo overexpression (Test) strain by transforming *C. trachomatis* with the plasmid pMT1918, which expresses Euo under the control of a Tet-inducible promoter (Fig. 2A). We also generated a Control strain expressing mCherry under the control of Tet-inducible promoter instead of Euo (Fig. 2A). We then performed RNA-seq analyses to compare transcript levels in these strains in the presence of aTc inducer at 16, 28, 32, and 36 hpi (Fig. S2A). At 16 hpi, only *euo* was differentially transcribed, consistent with its induced overexpression. At 28 hpi, Euo overexpression decreased transcription of 29 genes by ≥1.3-fold (*P* ≤ 0.05) (Fig. 2B; Table S2) and increased transcription of 86 genes (excluding *euo*) by ≥1.3-fold (*P* ≤ 0.05) (Fig. 2D; Table S2). At 32 hpi, 46 genes were downregulated (Fig. 2B) by ≥1.3-fold (*P* ≤ 0.05), and 145 genes (excluding *euo*) were upregulated by ≥1.3-fold (Fig. 2D). After 36 h of Euo overexpression, 23 genes were downregulated (Fig. 2B) (*P* ≤ 0.05), and 21 genes (excluding *euo*) were upregulated (Fig. 2D).

We then integrated our DIP-seq and RNA-seq data to identify genes that had an associated DIP-peak and were also differentially transcribed in response to Euo overexpression. We classified genes into six classes for each time point: (i) peak-associated AND upregulated genes, (ii) peak-associated AND downregulated genes, (iii) peak-associated AND constitutively expressed genes, (iv) NOT peak-associated AND downregulated genes, (v) NOT peak-associated AND upregulated genes, and (vi) NOT peak-associated AND constitutively expressed genes (Fig. S2). The peak-associated genes were candidate Euo targets, especially if they were differentially transcribed in response to Euo overexpression.

The absolute size and relative proportions of these six gene classes depended on the time point of the RNA-seq analysis (Fig. S2B) . For example, among the peak-associated genes, 16 (1.8%) were downregulated and 20 (2.2%) were upregulated at 28 hpi. More genes were differentially expressed at 32 hpi (192 genes) compared to 28 hpi (116 genes) (Fig. S2B). Approximately 25% (15/59) of all the downregulated genes were common between 28, 32, and 36 hpi time points (Fig. 2B), and about half (28/59) of all the downregulated genes were associated with a DIP-peak (Fig. 2C). In contrast, ~14% (21/149) of all the upregulated genes were common between 28, 32, and 36 hpi time points (Fig. 2D), and ~22% (33/149) of all the upregulated genes were associated with a DIP-peak (Fig. 2E). We interpreted the RNA-seq data from 36 hpi with some caution because some host cells infected with the Control strain started to lyse at this late time point (Fig. 6G), which could affect the accuracy of the differential expression comparisons.

To determine the temporal profile of Euo-regulated genes, we performed an RNA-seq analysis on the Control strain at 16, 24, 28, 32, 36, and 42 hpi, without aTc induction. Transcript levels for downregulated genes with a DIP-peak were most abundant at late times (24 hpi) (Fig. 2F; Table S4). In contrast, transcript levels for the majority (24/33; 72.7%) of upregulated genes with a DIP-peak were most abundant at midcycle (20 hpi), while just over a quarter (9/33, 27.3%) had peak transcript levels at late times (Fig. 2G; Table S4). In a similar vein, transcripts of all downregulated genes without a DIP-peak were most abundant at late times (Fig. S3A; Table S4), while transcripts of most (98/117, 83.8%) upregulated genes not associated with a DIP-peak were abundant during midcycle, and only 19/117 (16.2%) were abundant at late times (Fig. S3B; Table S4). These data indicate that Euo overexpression correlated with decreased transcription of late genes but increased transcription of some midcycle genes.

### The location of the Euo DIP-peak differs between downregulated and upregulated genes

**Fig 3:**
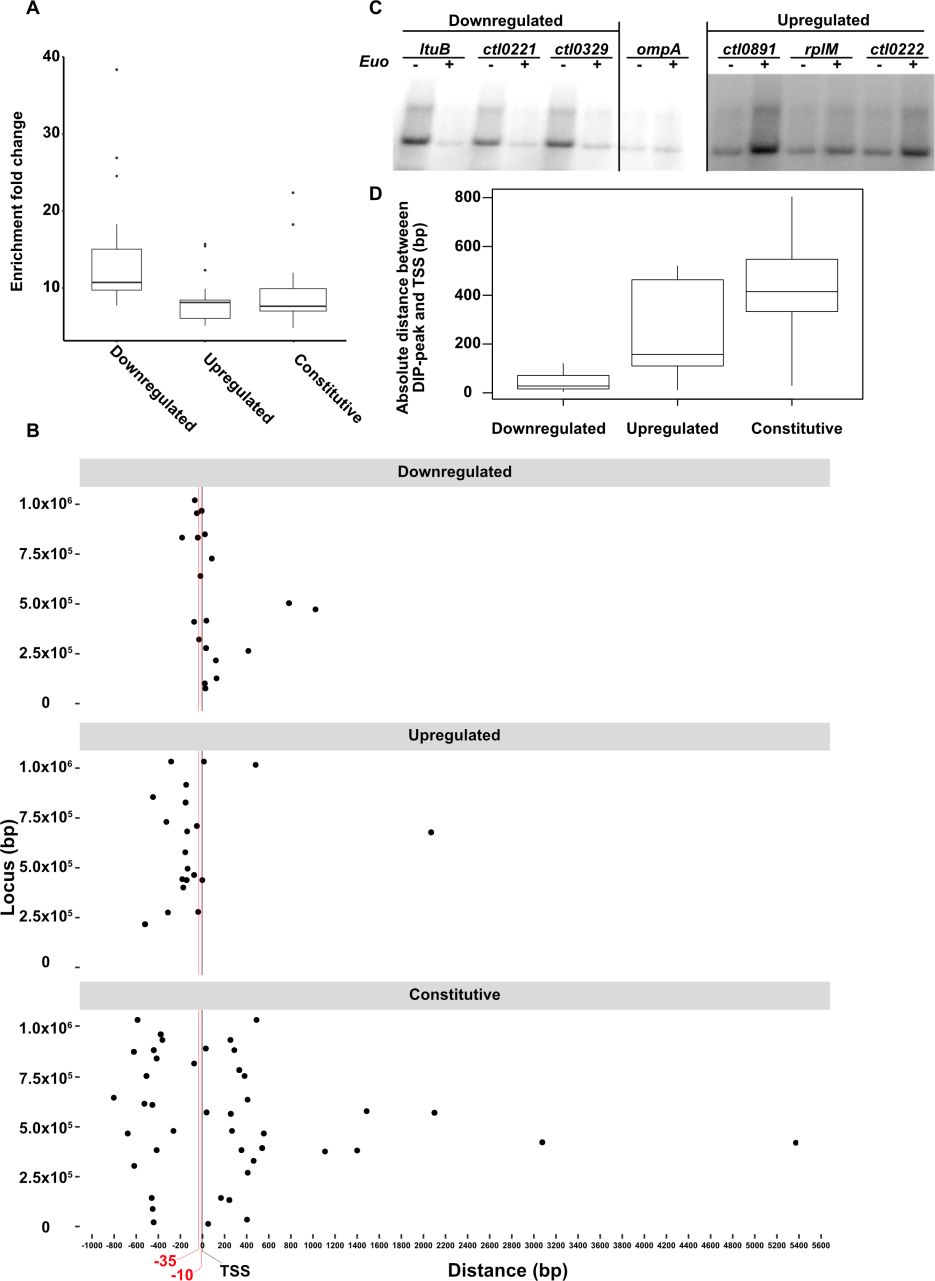
(A) Enrichment fold change between immunoprecipitated and mock (0 ng Euo) samples shows higher enrichment for DIP-peaks associated with downregulated genes compared to the peaks associated with constitutively expressed or upregulated genes. (B) Position of DIP-peaks with respect to the TSSs of associated genes (downregulated/upregulated) or nearest (constitutive) TSSs. DIP-peaks associated with downregulated genes overlapped the core promoter (located at −35 and −10 relative to the TSS), while those associated with upregulated genes were mostly within 200 bp upstream of the TSS (but upstream of the core promoter). DIP-peaks not associated with any differentially expressed gene were farther (>200 bp) upstream or downstream of the nearest TSS. (C) *In vitro* transcription of a representative set of downregulated, upregulated, and constitutively expressed promoter in the presence (+) or absence (−) of Euo demonstrates that Euo can directly repress or activate transcription. (D) Boxplot showing absolute distances between DIP-peaks and corresponding TSSs. Extreme outliers are not plotted for better resolution.

We next examined the relationship between the strength and location of Euo binding, as measured with our genome-wide Euo DIP-seq analysis, and the location of the promoter (see Materials and Methods). First, we used a bubble plot to visualize the relationship between the strength of Euo binding and differential expression. In general, downregulated genes were associated with stronger Euo binding (higher enrichment fold change, as shown by a larger bubble) compared to upregulated or constitutively expressed genes (Fig. S4). Downregulated genes as a group also had higher DIP enrichment which suggests stronger Euo binding (Fig. 3A). We also plotted the location of DIP-peaks relative to each TSS, as determined from Albrecht et al. (25). For the majority of DIP-peaks associated with downregulated genes, the center of the DIP-peak fell within the predicted promoter region (−35 to −10 relative to the TSS) (Fig. 3B, top panel). In contrast, the centers of the majority of DIP-peaks associated with upregulated genes were located upstream of their respective TSS, and most were within a distance of 200 bp (Fig. 3B, bottom panel). Centers of DIP-peaks that were not associated with a differentially expressed gene were scattered, without any obvious relationship with the location of their respective TSS (Fig. 3B, middle panel). The median absolute distance between DIP-peaks and corresponding TSSs was increased in the order of downregulated genes to upregulated genes to constitutively expressed genes (Fig. 3D).

We performed additional assays to verify two published (*ltuB*, *scc2*) (12) and two candidate target genes (*ctl0221*, *recB*) that had a DIP-peak and were differentially expressed with Euo overexpression, and four non-target genes (*ctl0473*, *recA*, *pyrH*, *folP*) that lacked a DIP-peak and were not differentially expressed (Fig. S5A). All four candidate target genes showed differential transcription by RT-qPCR (reverse transcription-quantitative polymerase chain reaction) (Fig. S5B) and produced an EMSA gel shift with recombinant Euo (Fig. S5C). By contrast, none of the four non-target genes showed differential transcription by RT-qPCR or an EMSA gel shift with Euo. The *omcA* promoter was used as a positive control for both RT-qPCR and EMSA experiments (Fig. S5B–D). These results demonstrate that Euo binding correlates with differential expression at the level of individual genes.

We also performed *in vitro* transcription assays using recombinant Euo (12). Recombinant Euo reduced transcription of promoters for three downregulated genes (*ltuB*, *ctl0221*, and *ctl0329*) but increased transcription of three upregulated genes (*ctl0891*, *rplM*, and *ctl0222*) (Fig. 3C; Fig. S5E). These promoters were selected due to the proximity of their DIP-peak center and TSS. Transcription of the *ompA* control promoter was not altered by Euo. Together these validation studies provide confirmatory evidence that Euo can repress or activate specific target genes.

### A single Euo binding site can regulate more than one target gene

**Fig 4:**
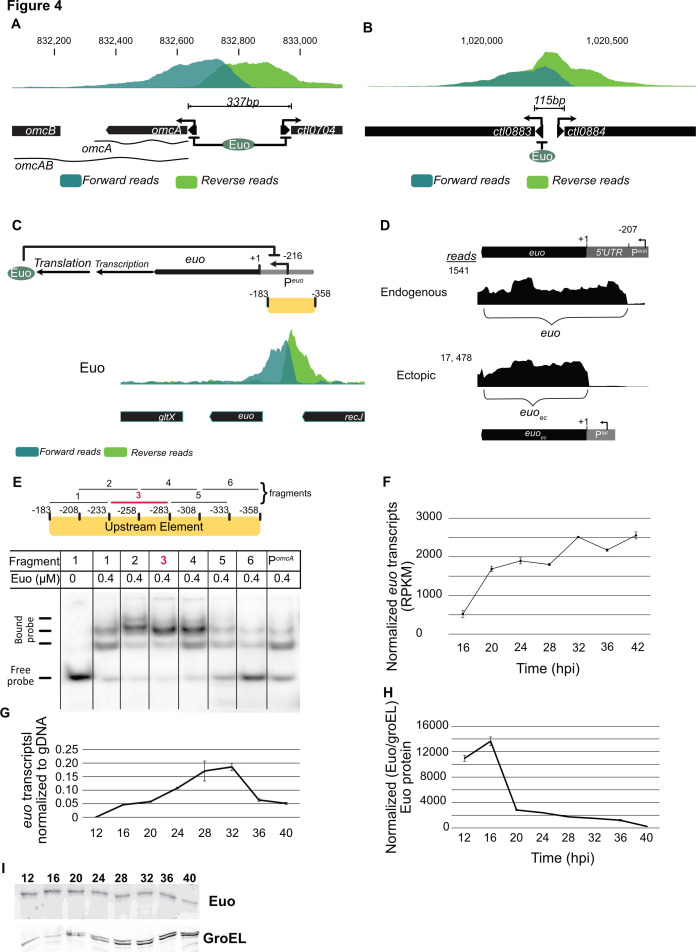
(A) Euo DIP-peak present in the 337 bp long intergenic region of *omcA* and *ctl0704*. Potential transcripts from the omcAB promoter are represented by wavy lines. (B) Euo DIP-peak present in the 115 bp intergenic region of *ctl0883* and *ctl0884*. (C) Schematic representation of Euo auto-regulation (top). Euo DIP-peak present in the intergenic region of *euo* and *recJ* (bottom). The center of the DIP-peak is located at −216 bp with respect to the *euo* start codon (+1). (D) Endogenous *euo* with a 207-bp 5′UTR identified by RNA-seq reads to the *C. trachomatis* genome. This 5′UTR is absent in ectopically expressed *euo* (*euo*_ec_). +1 represents the start codon of *euo,* and P*^euo^* represents its promoter region. −207 is the transcription start site. The tracings show reads mapped to *euo* for the Control (endogenous) vs the Test (ectopic) strains. (E) EMSA. Six 50 bp oligonucleotides (with 25 bp overlap) spanning the DIP-peak (position: −183 to −358 relative to start codon) *euo* were synthesized and radiolabeled. “Free probe” is the radiolabeled DNA used in the reaction, and “Bound probe” indicates the retarded DNA probe. (F) *euo* Transcripts normalized to gene-length, and total mapped reads (RPKM [reads per kilobase per million]) measured in the Control strain at 16, 20, 24, 28, 32, 36, and 42 hpi by RNA-seq. (G) *euo* mRNA measured in the Control strain by RT-qPCR and normalized to bacterial gDNA. (H) Protein level of Euo normalized to GroEL measured in the Control strain throughout the developmental cycle. (I) Western blot showing temporal expression pattern of Euo and GroEL.

Our combined DIP-seq and RNA-seq analysis provided examples of how Euo binding can simultaneously regulate more than one gene. For example, we detected an Euo DIP-peak encompassing the intergenic region between the divergently transcribed genes, *omcA* and *ctl0704* (Fig. 4A), which raised the possibility that Euo could regulate one or both genes. Furthermore, *omcA* is transcribed by itself as a 0.5-kb *omcA* transcript, and together with its downstream gene *omcB* as a polycistronic 2.3-kb *omcAB* transcript (*omcA* and *omcAB* in Fig. 4A) (25, 28). We used the differential expression data from our Euo overexpression RNA-seq analysis to distinguish among these possibilities. Euo overexpression caused downregulated transcription of *omcA*, *omcB,* and *ctl0704* (Table S2). Thus, it appears that Euo represses all three genes. In contrast, there was an Euo DIP-peak in the 115-bp intergenic region between the divergently transcribed genes *ctl0883* and *ctl0884* (Fig. 4B), but only *ctl0883*, and not *ctl0884*, was downregulated in response to Euo overexpression (Fig. 4B). These findings suggest that Euo selectively represses *ctl0883* but not *ctl0884*. Thus, the presence of an Euo binding site upstream of a transcription start site does not always mean that the gene is regulated by Euo.

### Euo regulates its own expression through a negative feedback loop

Unexpectedly, we discovered a strong DIP-peak upstream of the *euo* gene (Fig. 4C). To examine if Euo regulates its own expression, we took advantage of the 5′-UTR region of endogenous *euo,* which is not present in the plasmid-based copy of *euo* that was used for overexpression (Fig. 4D). Euo overexpression increased overall e*uo* transcript levels when we used primers to the Euo coding region, which do not distinguish between endogenous and ectopic *euo* mRNA (Fig. S1F). However, when we used primers to the 5′-UTR region that is specific for endogenous *euo*, there was a threefold decrease in transcription of endogenous *euo* compared to the Control strain (Fig. S1F). This result is consistent with the decrease in endogenous *euo* transcript levels measured by RNA-seq after 32 h of Euo overexpression (Table S2). These data suggest that *euo* represses its own transcription.

We performed EMSA experiments to confirm and locate the Euo binding site for *euo*. We generated six 50 bp fragments with 25 bp overlaps to span the width of the *euo* DIP-peak, between −183 and −358 bp upstream of the *euo* start codon. Recombinant Euo bound and generated a gel shift for all six DNA fragments, but the most intense signal was observed for fragments 2, 3, and 4 (Fig. 4E), which share a 50-bp region between −233 and −283 bp that appears to be important for Euo binding. This region contains the AT-rich sequence- AC**AAAAAA**CTTATTAATCAAGTGGTTTGTT**AAAAA**TAAATGCTA**TTTTT**G and lies 14 bp upstream of the *euo* transcription start site, in the vicinity of the *euo* promoter. This location suggests that Euo binding represses transcription by blocking access of RNA polymerase to the *euo* promoter. This region also contains a poly-T and two poly-A tracts (bolded above), consistent with our observation that most regions containing Euo DIP-peaks have poly-A tracts.

As Euo appears to be a temporal regulator of chlamydial gene expression, we examined the temporal pattern of its endogenous expression. Using HeLa cells infected with *C. trachomatis,* we quantified *euo* transcript levels by RNA-seq and RT-qPCR and found that transcript level increased between 16 and 32 hpi (Fig. 4F and G). In contrast, Euo protein levels, as measured by Euo IP and Western blots, decreased after 16 hpi (Fig. 4H and I). These contrasting results suggest that Euo levels may be subject to post-translational regulation.

### Overexpression of Euo causes developmental defects in *C. trachomatis*

**Fig 5:**
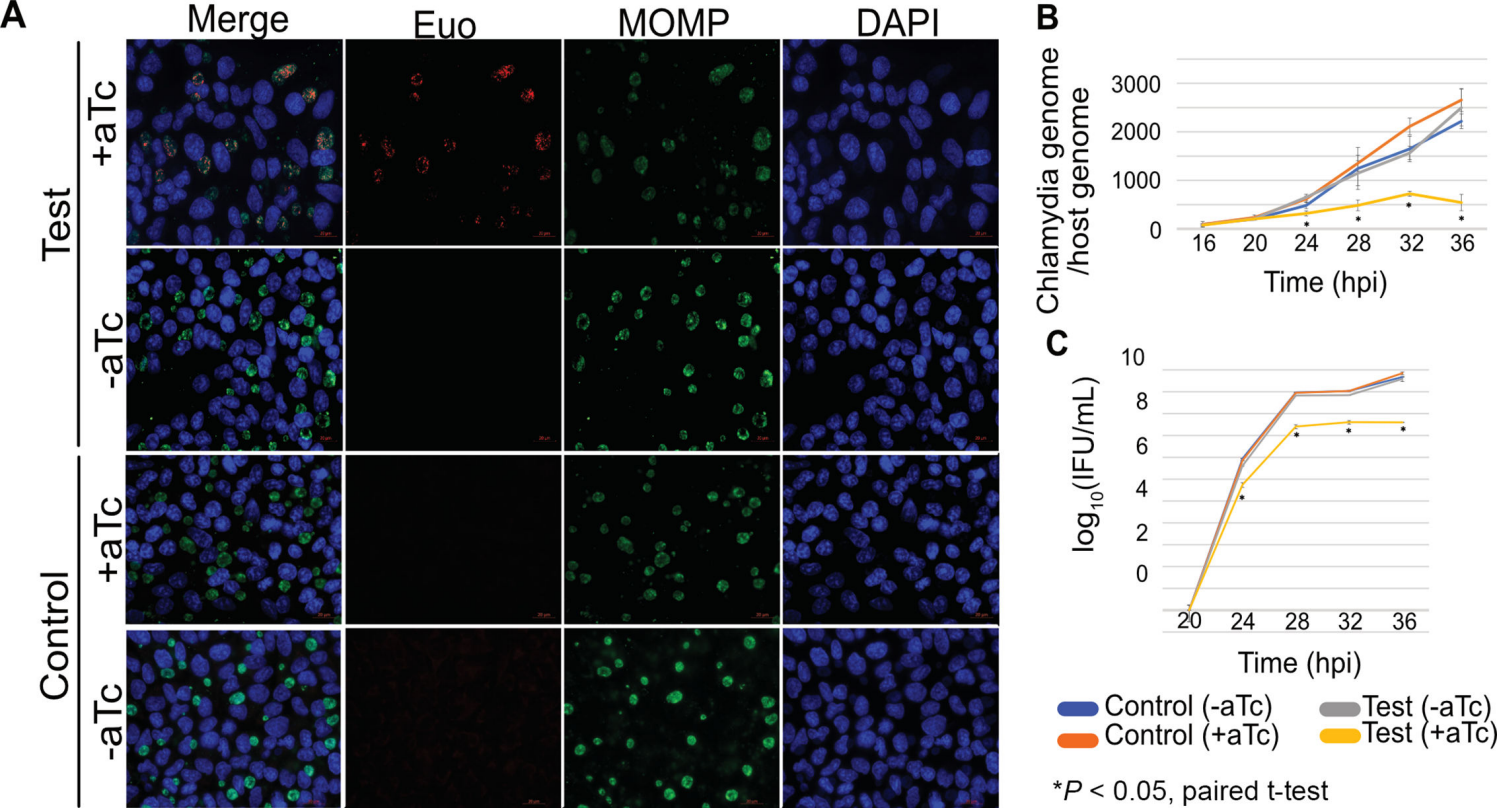
(A) Tetracycline-inducible expression of euo in *C. trachomatis*. HeLa cells were infected with the Test and the Control strains for 28 hpi, aTc hydrochloride was added to induce the expression of euo from 0 hpi. All Euo-stained (red) images were taken at the same exposure. Green staining indicates the expression of MOMP. DAPI (4',6-diamidino-2-phenylindole) was used to stain the DNA of both HeLa cells and *C. trachomatis*. Scale bar: 20 μm. (B) Quantitative PCR was used to quantify chlamydia copy number relative to host genomic DNA. Shown are the means ± SE (*n* = 2) for 20, 24, and 28 hpi. (C) Progeny assay. HeLa cells were infected with the Test or the Control strains. At 20, 24, 28, 32, and 36 hpi, cells were lysed in SPG buffer and used to re-infect a fresh monolayer of HeLa cells, and inclusions were counted. Data plotted as mean ± SE (*n* = 2). +aTc and −aTc indicate induced and uninduced samples, respectively. Test, Euo overexpressing *C. trachomatis* strain; Control, mCherry expressing *C. trachomatis* strain.

To measure effects of altered Euo levels on the chlamydial developmental cycle, we expressed His-tagged Euo and a GFP reporter under the control of an Euo-regulated promoter (P*^omcAB^*-GFP) (12). We confirmed that the His tag did not affect the DNA binding activity of purified Euo (Fig. S5D) (12) and confirmed ectopic overexpression of Euo by immunofluorescence and Western blot analysis (Fig. 5A; Fig. S5F and G). Induction with aTc increased *euo* transcript levels by sixfold, as measured by RT-qPCR, compared to the Control strain (Fig. S5H). Euo overexpression also decreased transcripts from the P*^omcAB^*-GFP reporter by 10-fold but had no effect on the expression of *fliF*, which is an Euo-independent constitutive gene (6) (Fig. S5H). Euo overexpression also decreased protein levels of the Euo-regulated late gene *scc2* at late times (Fig. S5I).

Euo overexpression reduced the number of chlamydiae per host cell by approximately threefold at 28, 32, and 36 hpi, as measured by qPCR (Fig. 5B), suggesting reduced chlamydial replication. We performed progeny assays at 24, 28, 32, and 36 hpi and observed that Euo overexpression reduced infectious progeny by more than 10-fold at all the time points (Fig. 5C). These results demonstrate that overexpression of Euo had deleterious consequences on the *C. trachomatis* developmental cycle.

### Overexpression of Euo causes defects in RB division and RB-to-EB conversion

In a complementary approach, we used transmission electron microscopy (TEM) to visualize and quantify the effects of Euo overexpression in *C. trachomatis*-infected HeLa cells. Euo overexpression decreased average inclusion size from ~125 to ~75 µm^2^ by EM (electron microscopy) (Fig. S6A), similar to a 40% reduction (~50 to ~30 µm^2^) (Fig. S6D) observed by immunofluorescence microscopy. These observations suggest that Euo overexpression has an inhibitory effect on inclusion growth.

**Fig 6:**
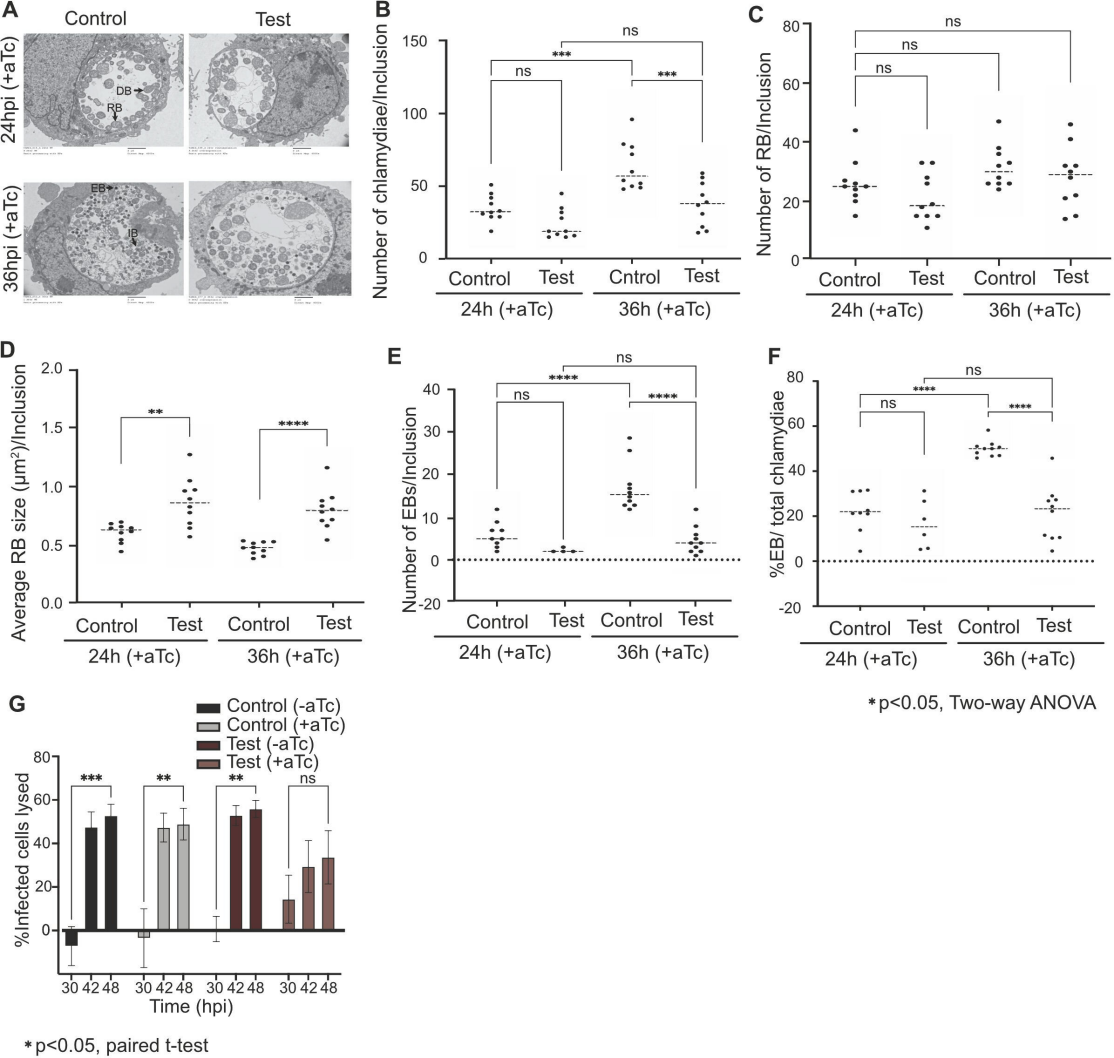
(A) Transmission electron microscopy analysis of the effect of Euo overexpression (Test). HeLa cells were infected with the Test or the Control strain and induced with aTc from 0 hpi. At 24 and 36 hpi, infected cells were processed for electron microscopy analysis. RB is reticulate body, DB is dividing RB, IB is intermediate body, and EB is elementary body. A total of 10 inclusions per strain were counted for each metric. (B) Total number of bacteria/inclusion. (C) Number of RBs/inclusion. (D) Average size (cross-sectional area) of RBs in each inclusion. (E) Number of EBs/inclusion. (F) Number of EBs as percentage of total bacteria in each inclusion. (G) Percentage of infected host cells at 24 hpi lysed at 30, 42, and 48 hpi in the Test and the Control strain. +aTc and −aTc indicate induced and uninduced samples, respectively. Test, Euo overexpressing *C. trachomatis* strain; Control, mCherry expressing *C. trachomatis* strain.

Euo overexpression also had effects on chlamydial developmental forms. Between 24 and 36 hpi, the total number of chlamydiae per inclusion increased in the Control strain, but there was no significant increase in the Euo overexpressing strain (Fig. 6) The mean number of RBs per inclusion did not change significantly between 24 and 36 hpi (Fig. 6C) in either the Euo overexpressing (Test) or Control strains. However, RBs were larger and more heterogeneous in size when Euo was overexpressed (Fig. 6D, see Fig. S6B for individual RB sizes). Euo overexpression also caused a significant reduction in the mean number per inclusion of EBs (Fig. 6E) and IBs, which is an RB in the process of converting into an EB (Fig. S6C), as well as the number of EBs as a percentage of total bacteria per inclusion (Fig. 6F). We did not observe these effects when mCherry expression was induced in the Control strain, which indicates that our Euo overexpression phenotype is not a pleiotropic effect of protein overexpression (Fig. 5B and C; Fig. 6; Fig. S5J). These EM findings indicate that Euo overexpression causes defects in inclusion growth and lysis, RB replication, and RB-to-EB conversion.

We also noted that Euo overexpression had an effect on host cell lysis. We determined the percentage of host cells that had lysed by counting the number of inclusion-bearing HeLa cells at 30, 42, and 48 hpi compared to the number at 24 hpi, prior to host cell lysis. For Control cells, with or without aTc induction, and for uninduced Test cells, at least 50% of inclusion-bearing host cells at 24 hpi lysed between 30 and 42 hpi. However, when Euo was overexpressed, we no longer observed this increase in lysed host cells from 24 to 48 hpi (Fig. 6G). For these studies, we verified that *euo* transcripts were still detected after 36 h of Euo induction (Fig. S1G). These results show that Euo overexpression inhibited the lysis of *Chlamydia*-infected host cells.

## DISCUSSION

In this study, we investigated the transcription factor Euo and its regulon by combining two high-throughput techniques, DIP-seq paired with RNA-seq in the context of Euo overexpression. Separately, these approaches allowed us to identify *in vitro* Euo binding sites and putative Euo-regulated genes, respectively. In addition, by correlating these results, we were able to distinguish between the direct and indirect regulons of Euo. We also determined the temporal expression profile of Euo target genes with a time-course RNA-seq analysis and combined it with published genome-wide TSSs (25) to identify operons and investigate the location of Euo binding sites relative to the TSSs. This integration of three new and independent sets of genome-wide data confirmed the proposed role of Euo as a transcriptional repressor of late genes but also revealed that Euo can activate transcription of a subset of midcycle genes and control the expression of its own gene.

Prior to this study, there was an incomplete picture of the role of Euo as a transcriptional regulator. Putative Euo-regulated genes have been identified with *in vitro* studies that used reconstituted RNA polymerase and purified recombinant Euo to analyze individual promoters (12, 13, 15, 29). This targeted approach has been helpful in showing that Euo is a transcriptional repressor but has limitations for defining the full complement of Euo-regulated genes. Chromatin immunoprecipitation (ChIP)-seq studies in the *Chlamydia*-related bacterium *Waddlia chondrophila* have identified Euo binding sites (30)*,* although it is not known if this binding results in transcriptional regulation, and it is difficult to extrapolate these results to *C. trachomatis*, whose genome is less than half the size of *Waddlia*. Wurihan et al. reported that Euo overexpression in *C. trachomatis* caused differential transcription of 467 genes, which is half the total number of genes, but this RNA-seq analysis did not distinguish between directly and indirectly regulated genes (31). The advantage of our approach is that we were able to define and distinguish the direct and indirect regulons of Euo in *C. trachomatis* by correlating our genome-wide analyses of Euo binding and differential transcription of Euo target genes.

One compromise that we made was to use DIP instead of ChIP to identify Euo binding sites in the *C. trachomatis* genome. Despite multiple efforts, we were unable to use ChIP to successfully recover Euo-bound DNA fragments from *C. trachomatis*-infected cells. We tried anti-Euo antibodies and anti-His antibodies in a His-tagged Euo strain, but an unresolved problem with formaldehyde fixation appeared to interfere with the immunoprecipitation step. As an alternative approach, DIP allowed us to identify Euo binding sites in the *C. trachomatis* genome. One caveat is that DIP does not measure the effects of other factors, such as the plasmid-encoded protein Pgp4 (32), and transcription factors GrgA and HrcA (31) that may regulate Euo. We are continuing our efforts to get Euo ChIP to work in *C. trachomatis* because it will allow us to measure temporal changes in Euo binding and regulation during the developmental cycle.

Our Euo overexpression results showed similarities and differences with the Wurihan Euo overexpression study (33). That study reported 467 differentially expressed genes (221 upregulated/246 downregulated) after 4 h of Euo overexpression from 12 to 16 hpi but did not measure differential expression at later time points. In our study, Euo overexpression caused no detectable differential expression at 16 hpi, but 208 genes were differentially expressed at 28–36 hpi. The two studies identified 29 upregulated genes (excluding *euo*) and 12 downregulated genes in common. In the light of delayed lysis (Fig. 6G) due to *euo* overexpression, a difference between the two studies is that Wurihan et al. (33) identified three genes involved in the inclusion stability (*ctl0481*/*cpoS*, *ctl0639*) and extrusion (*ctl0480*) (34), but these genes were not differentially expressed in our differential RNA-seq experiments. The reasons for the discordant effects of Euo overexpression on the other genes are not clear. For the 16 hpi time point, it is possible that we did not detect significant differential expression because of low sequencing coverage (see Table S3 for a detailed comparison with Wurihan et al.) (35). However, the ability of our Euo overexpression to cause differential expression at 28–36 hpi suggests that endogenous Euo levels were limiting at these late times and no longer regulating target genes. Thus, these later time points appear to be the most suitable for measuring the effects of higher Euo levels on the expression of its target genes. These data provide the first experimental evidence that the timing of Euo repression is temporally controlled in chlamydiae and suggest that derepression occurs at around 28–32 hpi, which is the time of late gene expression and RB-to-EB conversion (3, 6).

This study provides new insight into the role of Euo in late gene regulation. Previously, Euo had been shown to bind and repress transcription of four late promoters transcribed by σ^66^ RNA polymerase, which is the major form of chlamydial RNA polymerase (12, 13). Our analysis confirmed four of these putative Euo-regulated σ^66^ genes (*omcAB*, *scc2*, *ltuB;* Fig. S1H; Table S2) as direct Euo targets. However, we could not validate an additional gene, *sctU* (*cdsU*), as an Euo target as it did have a DIP-peak and was not differentially expressed (Table S2). Euo has also been reported to repress six genes (*hctB*, *tsp*, *ctl0508* [*tlyC_1*], *dnaK*, *pgk,* and *ctl0613* [*bioY*]) that are transcribed by σ^28^ RNA polymerase, which is an alternative form of chlamydial RNA polymerase (13, 36, 37) (Fig. S1I) (Table S2). However in our analyses, none of these genes had a DIP-peak (Table S2). One possible explanation is that the previous studies used EMSA to measure Euo binding to a single promoter region at a time, whereas DIP measures Euo binding in competition with all potential binding sites in the genome. Thus the previous Euo studies of σ^28^-regulated genes may have measured weak Euo binding that is not biologically meaningful.

Our study identified the subset of late genes that is repressed by Euo. These target genes include genes with EB-related roles, such as *omcA* and *omcB,* which encode EB-specific outer membrane proteins (38), genes involved in EB entry into a new host cell, such as *copB*, *copD, scc2* (39 – 42), and *tarP* (43 – 45)*,* and energy metabolism (*glgA*) (46, 47). However, not all late genes appear to be regulated by Euo, and we estimate that about half of late genes are direct Euo targets. Thus, Euo is an important regulator of late gene expression, but it is likely that there are additional mechanisms to control the transcription of late genes.

A new finding from this study is the identification of a subset of midcycle genes that are also regulated by Euo. Euo has been studied as a transcriptional repressor, but we identified a large number of DIP-peak-associated genes that were upregulated (Fig. 2D and E) by overexpression of Euo and had a midcycle expression pattern (Fig. 2G). DIP-peaks associated with these genes were predominantly located within 200 bp upstream of the putative promoters (Fig. 3B). Strikingly, 16 of the midcycle genes regulated by Euo are involved in maintaining ribosomal structure and rRNA binding (Table S2). An additional three genes encode translation initiation/termination factors, and two genes encode RNA polymerase subunits. Thus in midcycle, Euo appears to upregulate a subset of genes encoding the protein translation machinery. Several of the genes found to be upregulated due to Euo overexpression in this study were also reported by Wurihan et al., albeit at 16 hpi (Table S3).

The expanded role of Euo both as a transcriptional repressor and an activator is novel for *Chlamydia* but not unprecedented. For example, the bacterial transcription factor Fur (ferric uptake regulator) functions as a repressor when its operator overlaps the promoter but activates transcription when it binds ~100 bp upstream of the TSS (48, 49). We found a similar pattern in which Euo DIP-peaks were located in the vicinity of the promoter for Euo-downregulated genes but in a wider 200 bp region upstream of the TSSs for Euo-upregulated genes. More accurate annotation of TSSs will reduce errors in the measurement of TSS-to-DIP-peak distances. This new model of Euo both as a repressor of late genes and an activator of midcycle genes can be further tested with *euo* knockdown or knockout approaches.

There is no prior evidence that Euo regulates its own transcription, although there is ample precedent for auto-regulation of bacterial transcription factors as a negative feedback mechanism (50, 51). In our studies, Euo bound and repressed its own promoter, as shown by an Euo DIP-peak and decreased transcript levels with Euo overexpression. However, *euo* has a different expression pattern from other negatively regulated Euo targets, which are upregulated at 24 hpi, whereas *euo* is an early gene that is transcribed by 1 hpi (6, 14). However, we measured a similar threefold increase in *euo* transcript levels by RNA-seq and RT-qPCR between 16 and 32 hpi, consistent with the upregulation of Euo-repressed genes. The significance of the decrease in *euo* transcript levels detected by qPCR but not by RNA-seq at very late times (after 32 hpi) is not clear. This discordance could be due to the normalization method as qPCR *euo* transcript levels were normalized to bacterial gDNA while RNA-seq transcript levels were normalized to total mapped reads.

The importance of controlling Euo expression was underscored by our Euo overexpression studies, which showed that increased Euo levels inhibited RB-to-EB conversion and increased RB size (Fig. 6D; Fig. S6), providing evidence that it is important to control Euo expression levels during the developmental cycle. It is possible that these two phenotypes are linked because RB size has been proposed as an intrinsic signal that controls RB-to-EB conversion (3, 4). In this size control model, RBs are hypothesized to only convert into EBs when they reach a small threshold size, through multiple rounds of replication. Thus, it is possible that Euo could promote RB-to-EB conversion by regulating genes involved in RB size control. Alternatively, Euo may regulate the balance between RB replication and RB-to-EB conversion through its dual functions as a transcriptional repressor and activator. Our data showed that Euo overexpression decreased total chlamydial number (Fig. 5B), which was manifested as similar numbers of RBs but decreased EBs and IBs (Fig. 5C; Fig. 6; Fig. S6C). Thus, Euo overexpression may have prolonged the activation of midcycle target genes, favoring RB replication while extending the repression of late target genes and preventing RB-to-EB conversion.

From our findings, we propose a model in which Euo functions as an important regulator of chlamydial developmental gene expression. When RBs are dividing in midcycle, Euo binds and represses promoters of a subset of late genes to prevent their premature expression, while activating a subset of midcycle genes that may be involved in RB growth and replication. Euo also represses its own gene so that Euo expression levels are homeostatically controlled through negative feedback. At late times, Euo binding is abrogated, which leads to derepression of late target genes to mediate RB-to-EB conversion and decreased activation of midcycle target genes. The mechanism of derepression has not been defined, but our finding of decreased levels of Euo protein, but not transcripts, at late times suggests that it may be mediated by decreased translation or proteolysis of Euo.

In conclusion, this study expands the role of Euo as a temporal regulator of transcription during the *C. trachomatis* developmental cycle. We have identified the Euo regulon and subdivided it into late genes that are repressed, and a subset of midcycle genes that are activated, by Euo. These findings indicate that Euo is involved in regulating genes from two of the three major temporal classes of chlamydial genes, as well as its own gene, which is in the third major temporal class. The role of Euo as a repressor of late genes suggests that Euo may be involved in controlling the timing of RB-to-EB conversion. In addition, the new finding that Euo is also a transcriptional activator of a subset of midcycle genes suggests that it also plays a role in RB growth and replication. With its multiple roles in temporal gene regulation, Euo would make an attractive therapeutic target for interrupting the chlamydial developmental cycle.

## Data Availability

The sequencing data for ChIP-seq and differential expression RNA-seq discussed in this publication have been deposited in NCBI’s Gene Expression Omnibus (52) and are accessible through GEO Series accession numbers GSE202414 and GSE202415, respectively. Access to sequencing data for time-course RNA-seq can be requested privately. DIP-seq mapping and peaks are available on UCSC Genome Browser .

## AFTER EPUB

*[This article was published on 11 August 2023 with an error in Fig. 6. The figure was corrected in the current version, posted on 19 September 2023.]*

## SUPPLEMENTAL MATERIAL

Fig. S1 mbio.00465-23-s0001.tif Euo pull-down and DIP-seq.

Fig. S2 mbio.00465-23-s0002.tif Differentially expressed genes.

Fig. S3 mbio.00465-23-s0003.tif Expression profile of indirectly regulated genes.

Fig. S4 mbio.00465-23-s0004.tif Combined DIP-seq and RNA-seq bubble plot.

Fig. S5 mbio.00465-23-s0005.tif Binding of Euo at the promoters of *euo* sensitive genes.

Fig. S6 mbio.00465-23-s0006.tif EB and IB counts.

Table S1 mbio.00465-23-s0007.xlsx Number of reads.

Table S2 mbio.00465-23-s0008.xlsx Time point wise RNA-seq and DIP-seq.

Table S3 mbio.00465-23-s0009.xlsx Comparison with Wurihan et al.

Table S4 mbio.00465-23-s0010.xlsx Heatmap data.

ASM does not own the copyrights to Supplemental Material that may be linked to, or accessed through, an article. The authors have granted ASM a non-exclusive, world-wide license to publish the Supplemental Material files. Please contact the corresponding author directly for reuse.
