# Supplementary figures and images for: Euo is a developmental regulator that represses late genes and activates midcycle genes in Chlamydia trachomatis
Source: mBio. 2023 Aug 11;14(5):e00465-23. doi: 10.1128/mbio.00465-23 (PMC10653925; doi:10.1128/mbio.00465-23)

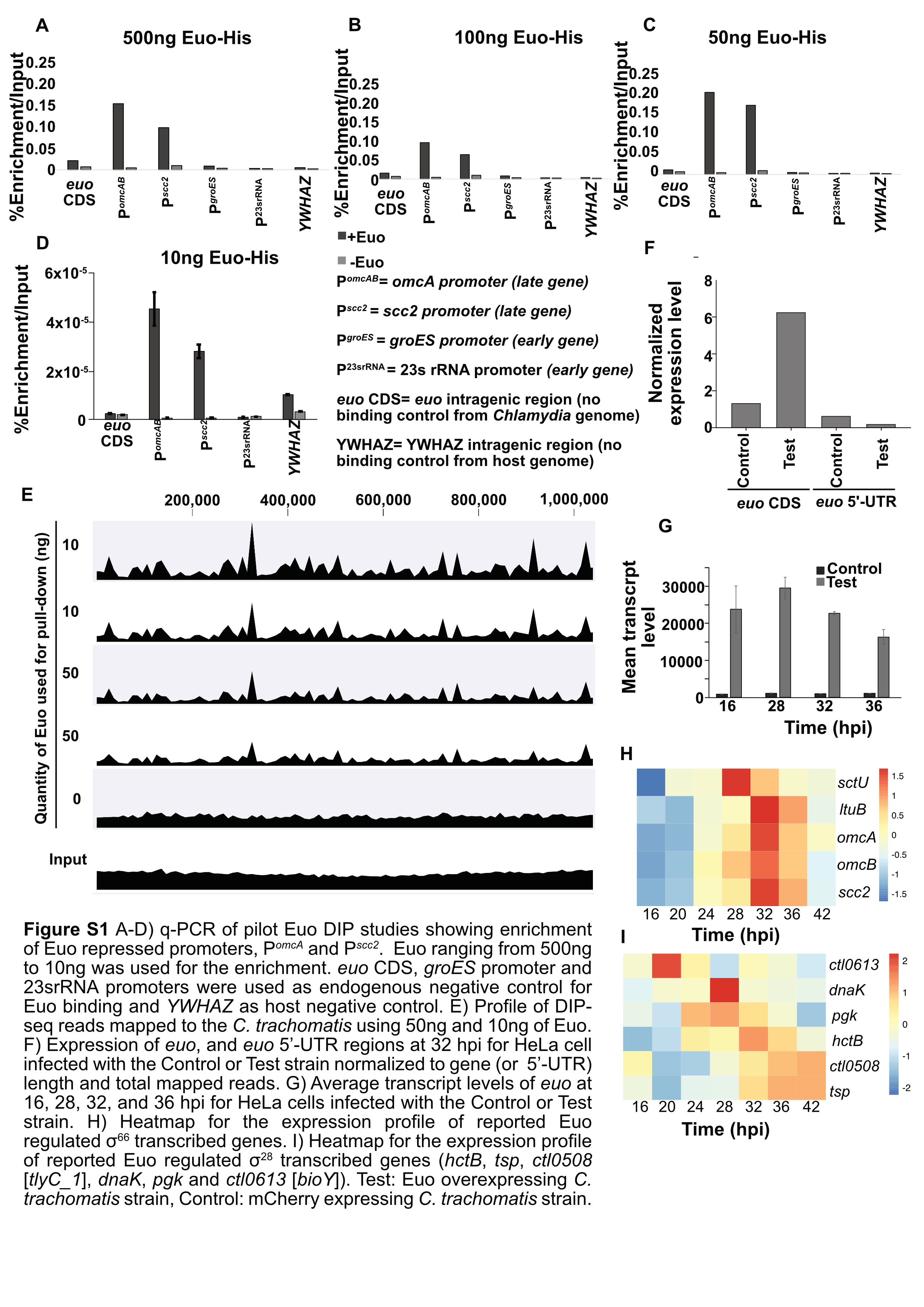

Supplement: Fig. S1 — Euo pull-down and DIP-seq. [file mbio.00465-23-s0001.tif]

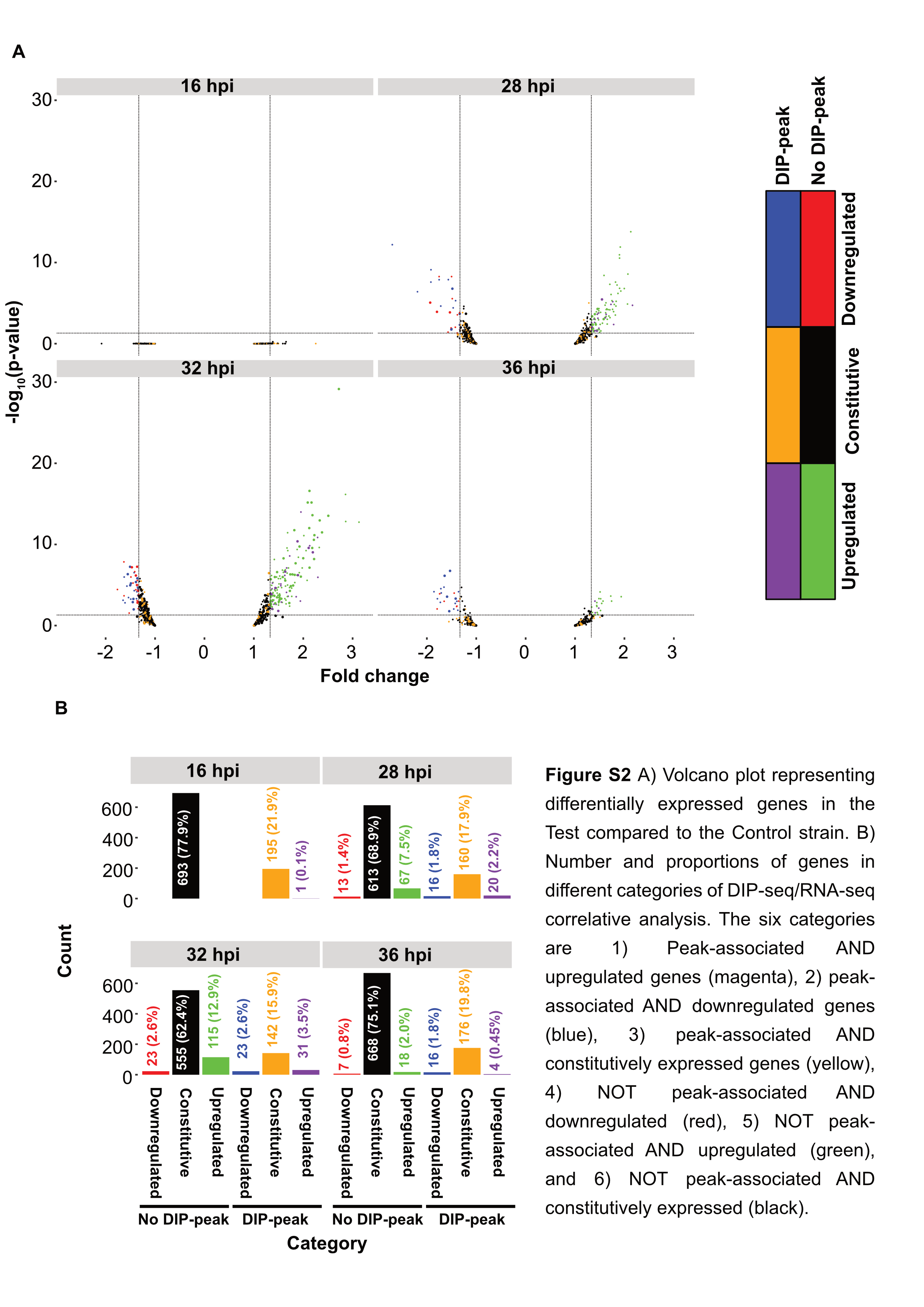

Supplement: Fig. S2 — Differentially expressed genes. [file mbio.00465-23-s0002.tif]

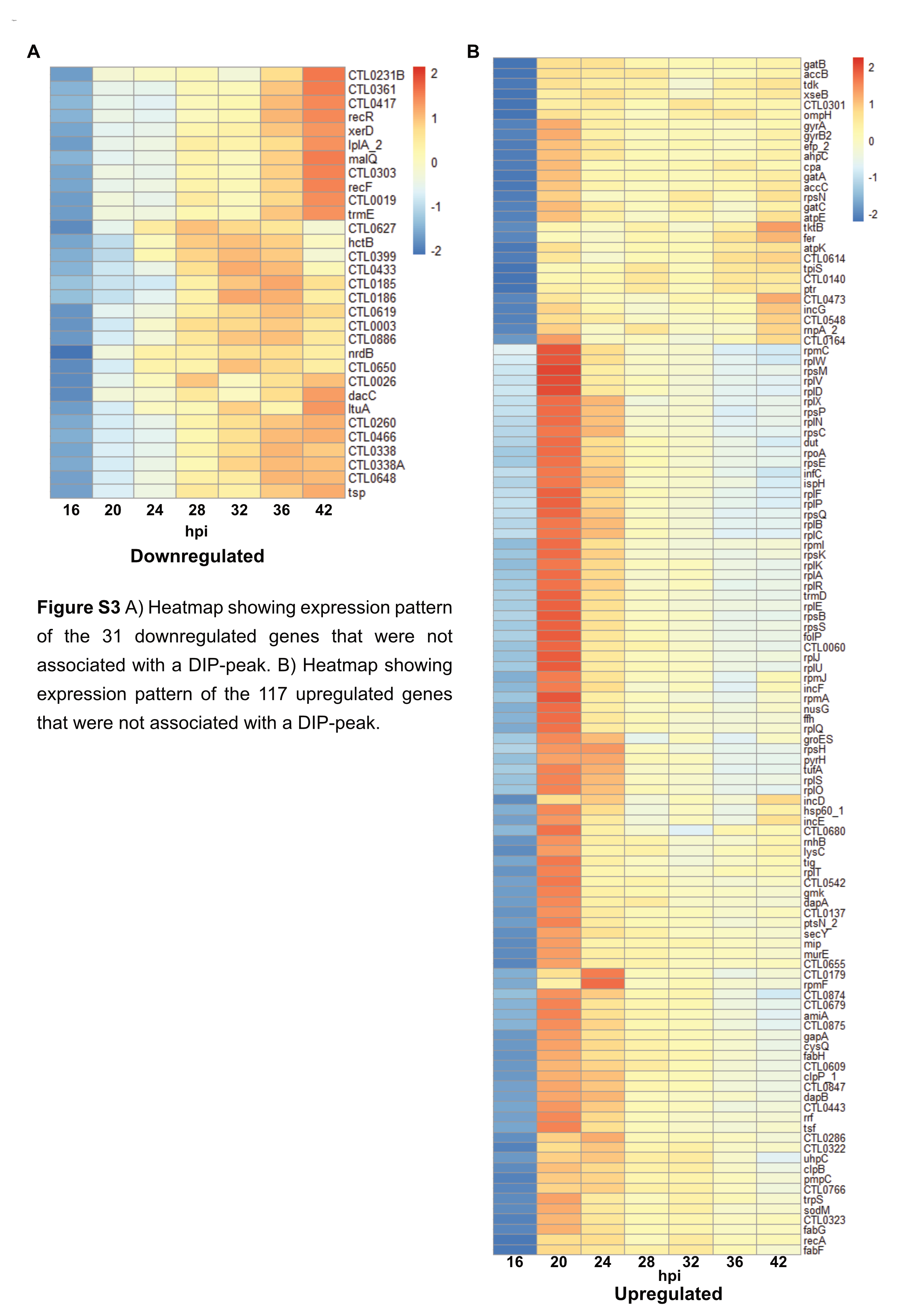

Supplement: Fig. S3 — Expression profile of indirectly regulated genes. [file mbio.00465-23-s0003.tif]

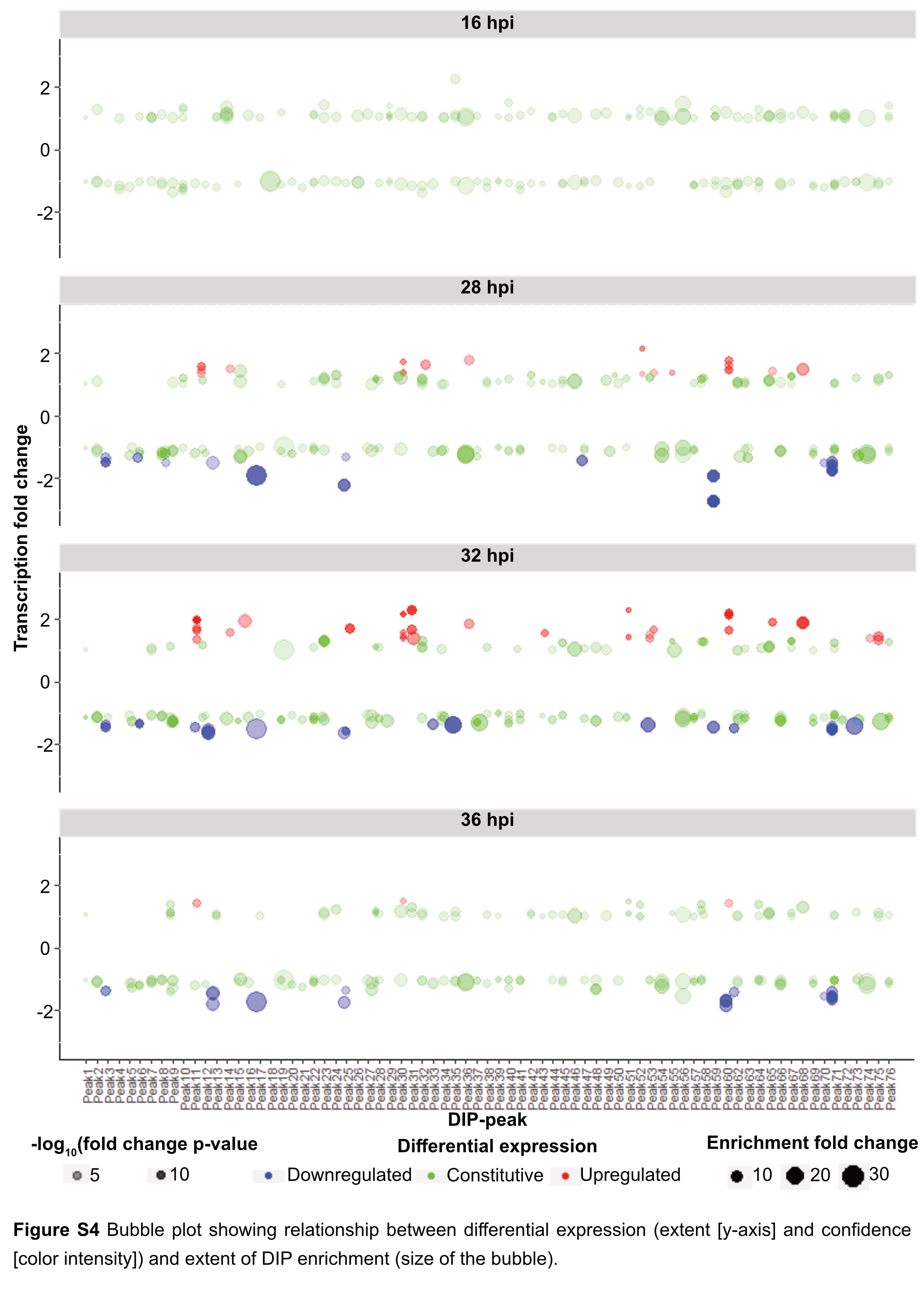

Supplement: Fig. S4 — Combined DIP-seq and RNA-seq bubble plot. [file mbio.00465-23-s0004.tif]

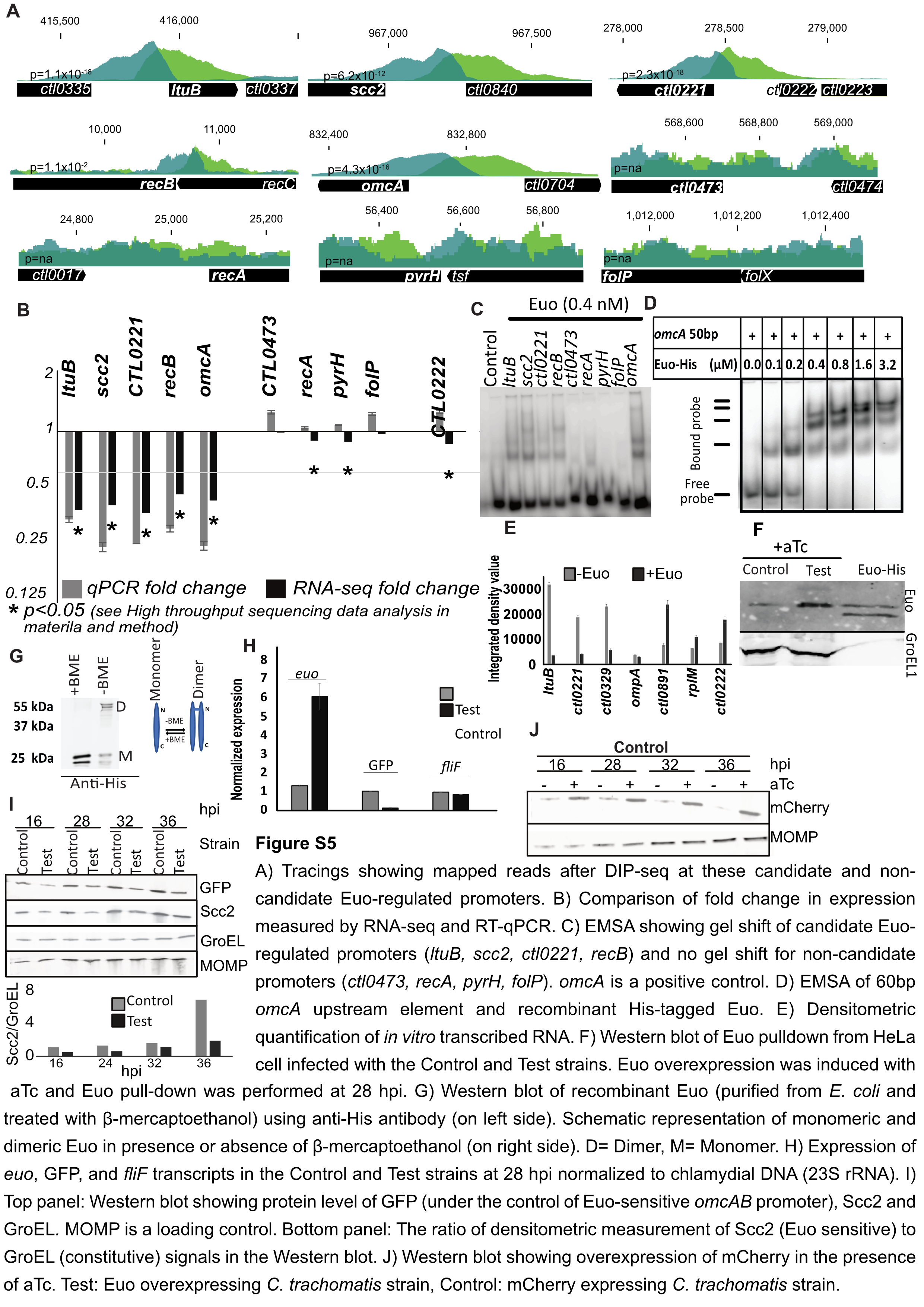

Supplement: Fig. S5 — Binding of Euo at the promoters of euo sensitive genes. [file mbio.00465-23-s0005.tif]

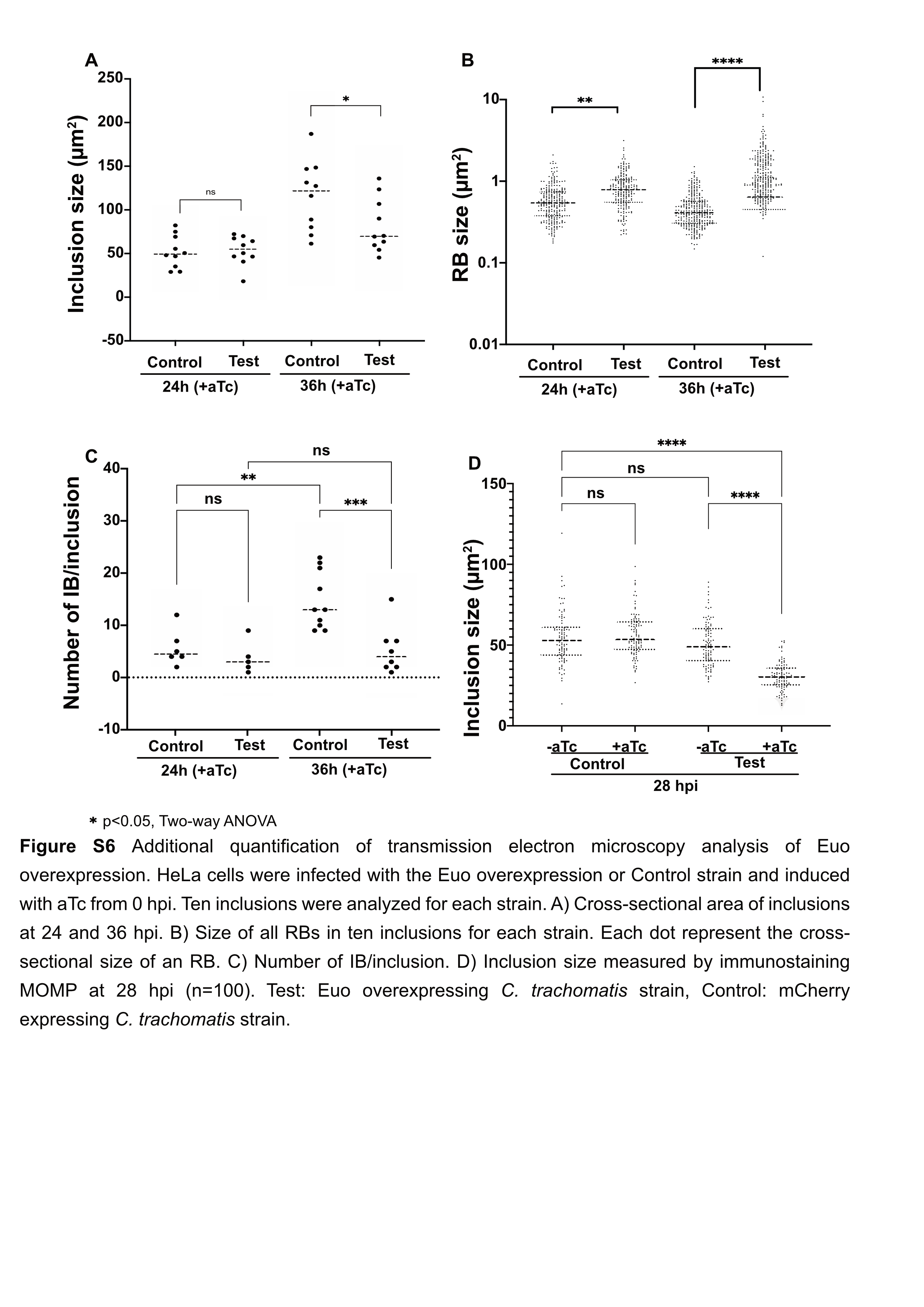

Supplement: Fig. S6 — EB and IB counts. [file mbio.00465-23-s0006.tif]
